# Mobile phone addiction and academic burnout: the mediating role of technology conflict and the protective role of mindfulness

**DOI:** 10.3389/fpsyt.2024.1365914

**Published:** 2024-03-04

**Authors:** Guang-Hui Yang, Xiao-Xuan Cao, Yan-Yan Fu, Ning-Dan Wang, Shuai-Lei Lian

**Affiliations:** ^1^ School of Education, Anyang Normal University, Anyang, China; ^2^ College of Education and Sports Science, Yangtze University, Jingzhou, China; ^3^ Key Laboratory of Adolescent Cyberpsychology and Behavior (CCNU), Ministry of Education, Wuhan, China

**Keywords:** mobile phone addiction, technology conflict, mindfulness, academic burnout, college students

## Abstract

With the rapid development of Internet technology, more and more college students are facing the threat of mobile phone addiction. However, the relationship and underlying mechanism between mobile phone addiction and academic burnout haven’t been explored in depth. This study proves the mediating role of technology conflict and the moderating role of mindfulness in the relation between mobile phone addiction and academic burnout. 752 college students were recruited to complete the questionnaire of mobile phone addiction, technology conflict, mindfulness and academic burnout. Results showed that mobile phone addiction was significantly and positively associated with academic burnout, and this relationship could be mediated by technology conflict. Besides, the direct effect of mobile phone addiction on academic burnout and the indirect effect of technology conflict in this link were moderated by mindfulness. Both these two effects are stronger for college students with lower level of mindfulness. Our findings enrich our understanding of how and when mobile phone addiction was related to academic burnout. Educational professionals and parents should take timely measure to the academic burnout of college students suffering from mobile phone addiction, particularly for those with lower level of mindfulness.

## 1 Introduction

Technology use has been acknowledged as a positive catalyst for societal progress, encouraging innovation, development, and the creation of value (1). Nevertheless, the outcomes of technology use may not consistently align with the intended goals. For instance, information technology overload, people were becoming overly dependent on technologies, and it even spawned the risk of individuals’ mobile phone addiction (2). Numerous studies have highlighted the adverse effects of mobile phone addiction on individuals, including psychological distress (3), irrational procrastination (4), and social isolation (5), particularly evident among college students. Not only do college students constitute the majority of mobile phone users, but they also exhibit a high prevalence of mobile phone addiction (6). The transition to college was characterized by multifaceted challenges encompassing emotional, social, and academic adaptation. It was crucial to emphasize that academic pursuits rank among the foremost responsibilities for college students. Unfortunately, existing research has predominantly focused on the detrimental effects of mobile phone addiction on emotional adaptation (3, 7, 8), behavioral adaptation (9), and sleep quality (10). Inadequate attention has been directed towards exploring the nexus between mobile phone addiction and the academic adjustment of college students. Consequently, our study aims to investigate the correlation between mobile phone addiction and college students’ academic burnout, elucidating the underlying mechanisms and individual differences.

Previous studies have extensively examined influencing factors of academic burnout from various perspectives, including family environment (11), academic adaptation (12–14), maladaptive behavior (15), and personality traits (16). With the rise of the Internet, researchers have also focused on the impact of Internet device use on individuals’ academic adaptation problems. Shen (17) demonstrated that Internet information seeking could improve academic self-efficacy, while Brate (18) showed that compulsive Internet use may lead to academic procrastination. In recent years, the widespread use of mobile Internet devices, such as smartphones, has increased the detection rate of mobile phone addiction. The negative impact of mobile phone addiction on college students’ academic adaptation problems has been revealed, affecting academic procrastination (19), academic performance (12), and learning engagement (14), either directly or indirectly. However, few studies have delved into the relationship between mobile phone addiction and college students’ academic burnout. The intrinsic mechanisms (how) and boundary conditions (for whom) of this relationship remain unclear. Therefore, it was imperative to explore the relationship between mobile phone addiction and college students’ academic burnout and unveil the intrinsic mechanisms and individual differences involved. The current study proposed a moderated mediation model that provided a comprehensive account of the relationship between mobile phone addiction and academic burnout from the perspectives of technology use experience (e.g., technology conflict) and individual traits (e.g., mindfulness).

### 1.1 Mobile phone addiction and academic burnout

Academic burnout manifests as a stress response stemming from students’ struggles to effectively manage academic stress, potentially resulting in a range of unfavorable outcomes (20). This phenomenon involves physical and mental exhaustion, academic alienation, and low achievement, constituting a three-dimensional challenge (21). Various studies have underscored the detrimental impact of academic burnout on individuals’ psychological and behavioral adaptation, contributing to issues such as depressive symptoms and heightened alcohol consumption (22, 23). In the contemporary digital era, where mobile phone addiction has emerged as a significant concern, it becomes crucial to recognize it as a potential and noteworthy predictor of academic burnout.

To begin with, mobile phone addiction was frequently associated with severe academic procrastination (19). The pleasure derived from swiping through mobile phones may result in reluctance to initiate study tasks for an extended period. Subsequently, when individuals decided to disengage from their mobile phones and commence studying, they may find the tasks challenging, requiring substantial time to grasp concepts not previously learned. This process proved mentally and physically exhausting, ultimately contributing to the development of academic burnout.

Besides, according to the elaborated intrusion theory of desire (24), the heightened cognitive processing experienced during cravings for mobile phones intensifies psychological desire, compelling individuals to persistently seek gratification for their psychological needs. In the context of mobile phone addiction, the continual craving for these devices may divert attention away from academic tasks, diminishing focus. Over time, individuals ensnared by mobile phone addiction may immerse themselves in the virtual world created by these devices, leading to a decline in interest and enthusiasm for learning—an occurrence consistent with the concept of academic alienation within the three dimensions of academic burnout.

Furthermore, numerous studies have unveiled the relationship between mobile phone addiction and negative emotions such as depression and anxiety (3). According to the broaden-and-build theory of positive emotions, positive emotions play a crucial role in helping individuals accumulate lasting personal resources and contribute to their self-realization (25). Conversely, persistent negative emotions could be detrimental to personal growth. Lingering negative emotions may lead individuals into a perpetual state of self-doubt and questioning, resulting in feelings of frustration and low academic achievement, ultimately inducing academic burnout.

In summary, we proposed hypothesis 1: mobile phone addiction may be positively associated with academic burnout.

### 1.2 Technology conflict as a mediator

Technology conflict, arising from an unhealthy response to new computer technology, has generated various negative effects on technical users (26). This maladaptive phenomenon manifests in two forms: technology-personal conflict and technology-academic conflict. Technology-personal conflict refers to health and physical problems that arise from the utilization of specific and general technologies. Likewise, technology-academic conflict encompasses academic challenges arising from the usage of specific pervasive technologies. Previous research has confirmed the connection between technical pressure and job burnout, highlighting the inducing effect of technology conflict on job burnout (27). Given that college students constitute the largest audience of information technology, the relationship between information technology and their learning and leisure time was closely intertwined. Some studies have identified technology conflict as the primary negative experience associated with mobile phones and other technological devices for college students (28). Unfortunately, few studies have delved into the correlation between technology conflict and college students’ academic adaptation problems. Therefore, this study aimed to build on prior research to explore the link between technology conflict and college students’ academic burnout and further investigate the mediating role of technology conflict in the relationship between mobile phone addiction and academic burnout.

While previous research may not have directly examined the relationship between technology conflict and academic burnout, indirect evidence suggested a connection. Firstly, individuals spending extensive time on technological devices often experience diminished attentional capacity (8). The negative impact of technology conflict, particularly reduced attentional capacity, may be associated with academic burnout (29). Besides, those addicted to technological devices may compromise their learning tasks (12). Excessive engagement with technological devices could result in a decline in academic performance and academic self-efficacy, contributing to academic burnout (30). Additionally, technology conflict could lead to the loss of certain resources, including physical health and academic performance. According to the resource conservation theory of burnout, individuals strived to maintain, protect, and establish their resources (31). When there was a potential loss or actual loss of these resources without effective preservation or replacement, individuals may experience fatigue. This fatigue could impair their ability to cope with academic tasks, ultimately leading to academic burnout.

Mobile phones, as technical devices, could trigger technology conflict through mobile phone addiction. Excessive use of mobile phones was an important trigger of technology conflict, and mobile phone addicts tend to spend long periods of time scrolling through their phones before bedtime, which could shorten their sleep time and reduce sleep quality (32). Meanwhile, excessive mobile phone use may impact cardiorespiratory fitness, exposing college students to a greater risk of health problems and inducing technology-personal conflict (33). Besides, according to resource limitation theory, when individuals’ attentional resources were focused on information or activities related to mobile phones, the resources at the disposal of the individual for learning would be reduced accordingly. At this time, individuals were more likely to have a series of academic adjustment problems and technology-academic conflicts. Hence, mobile phone addiction may be closely related to technology conflict that manifests itself as technology-personal conflict and technology-academic conflict. Social cognitive theory also suggests that individuals, behaviors, and environments interact with each other. Mobile phone addiction may induce a wide range of internal conflict experiences and external conflict scenarios (34), which could adversely affect individuals’ physical and psychological well-being, and lead to emotional adjustment problems such as academic burnout. Therefore, hypothesis 2 was proposed: technology conflict may act as a mediator in the relationship between mobile phone addiction and academic burnout.

### 1.3 Mindfulness as a moderator

Although mobile phone addiction has demonstrated a positive correlation with technology conflict and academic burnout, its impact may vary based on individual psychological traits. Therefore, it is essential to explore potential psychological traits that may moderate the relationship between mobile phone addiction and academic burnout. Additionally, to intervene with mobile phone-addicted individuals and mitigate the negative effects on their technology experience and academic adaptation, it was crucial to reveal the buffering effect of positive individual psychological traits, such as mindfulness, on the negative consequences of mobile phone addiction.

Mindfulness is characterized as a form of self-regulation wherein individuals direct their attention to the present moment in a conscious, non-judgmental, open, and receptive manner (35). Functioning as a positive psychological trait, mindfulness empowered individuals to consciously perceive their experiences without undue criticism, often acting as a significant buffer against other risk factors that may contribute to negative emotions and cognitive challenges. Prior research has indicated that mindfulness could play a moderating role in the relationship between mobile phone addiction and sleep quality (10). Additionally, a meta-analysis has demonstrated the effectiveness of mindfulness-based interventions in reducing depressive symptoms in adolescents (36). Furthermore, there was evidence suggesting a positive association between mindfulness and students’ academic performance (37). Thus, the current study aims to introduce mindfulness as a moderator to unveil its protective role in the relationship between mobile phone addiction and academic burnout, laying a theoretical foundation for intervention strategies in mobile phone-addicted individuals to prevent emotional and behavioral adaptation problems.

Previous research has consistently demonstrated that individuals with high levels of mindfulness exhibit the ability to pay attention to the present moment, accept their unhappiness, and maintain a non-judgmental attitude (35). This heightened mindfulness manifests in several ways that contribute to reducing the negative impact of mobile phone addiction on academic burnout. First, individuals with a high level of mindfulness could concentrate more effectively on their present tasks. This enabled them to allocate less attention to their mobile phones and, instead, focus on the current learning situation, where studying is the primary task (10). This enhanced focus on learning activities not only stimulated interest in the educational process but also reduced the risk of academic burnout, even when mobile phone addiction was present (10). Second, individuals with a high level of mindfulness were more likely to accept their maladaptive behaviors, reducing rumination over excessive mobile phone use. This acceptance played a crucial role in diminishing the negative impact of mobile phone addiction on individuals’ psychological states. Consequently, individuals with high levels of mindfulness could cope with academic challenges more effectively, leading to a lower likelihood of experiencing academic burnout (35). Moreover, individuals with a high level of mindfulness were better equipped to confront the adverse consequences of mobile phone addiction with a non-judgmental attitude. This positive mindset not only aided in dealing with various challenges but also made individuals more resilient in the face of academic difficulties. Consequently, they were less likely to encounter problems such as academic burnout due to the challenges associated with their studies. Furthermore, according to the mindfulness-based automation mechanism model (38), college students with a high level of mindfulness, after experiencing mobile phone addiction, could adeptly utilize mindfulness to refocus on specific goals. This enabled them to shift attention away from mobile phone use more swiftly, thereby reducing the perceived sense of resource deficiency and, consequently, experiencing less academic burnout.

Besides, mindfulness may act as a buffer in the relationship between mobile phone addiction and technology conflicts. Mindfulness enhanced individuals’ “de-centered” perspective and increased their capacity for spectator perception (39). Consequently, individuals with high levels of mindfulness could critically view their mobile phone addiction with a detached stance, facing it cautiously and with fewer emotional problems. This reduced the likelihood of technology-personal conflict. Additionally, individuals with high levels of mindfulness, despite a high risk of mobile phone addiction, could allocate attention to their ongoing academic tasks. This reduced the likelihood of neglecting studies due to mobile phone addiction, consequently decreasing the chances of experiencing technology-academic conflicts. Furthermore, individuals with high levels of mindfulness could accept their own mobile phone addiction, that is, they did not bring any subjective evaluation in the process of acceptance (40). Therefore, although individuals have a higher risk of mobile phone addiction, individuals with high mindfulness were less likely to fall into the emotional adjustment problems caused by mobile phone addiction, and fewer technology-personal conflicts occur. At the same time, individuals with high mindfulness also thought less about the adverse effects of mobile phone addiction, have more cognitive resources to deal with learning tasks related to their studies, and have fewer technology-academic conflicts. Moreover, based on previous studies on combined mediation and moderation models (10, 41), if technology conflict mediated the relationship between mobile phone addiction and academic burnout, and mindfulness simultaneously moderated the association between mobile phone addiction and academic burnout, the mediating role of technology conflict would be moderated by mindfulness. This led to a moderated mediation model involving technology conflict and mindfulness in the relationship between mobile phone addiction and academic burnout.

In summary, hypothesis 3 were proposed: mindfulness would have a negative moderating effect on mobile phone addiction and academic burnout, as well as on mobile phone addiction and technology conflict.

### 1.4 The present study

**Figure 1:**
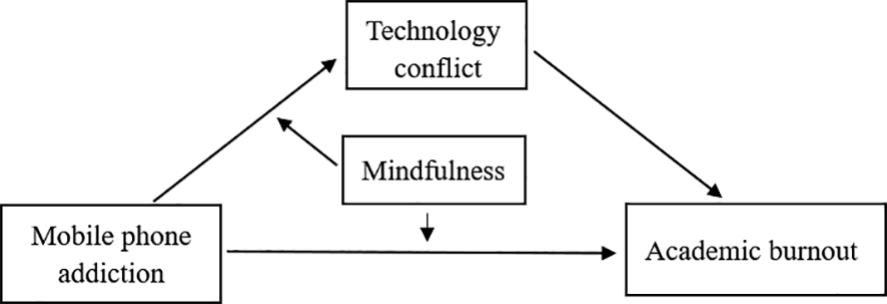
The proposed moderated mediation model.

To sum up, the present study aimed at examining whether the relation between mobile phone addiction and academic burnout among college students was mediated by technology conflict, and whether the effect of mobile phone addiction on academic burnout and the mediating effect of technology conflict were moderated by mindfulness. The integrated model proposed was outlined in **Figure 1**.

## 2 Method

### 2.1 Participants and procedure

In this study, 850 college students from the university located in Jingzhou, were recruited to take part in this survey with groups employing the convenient sampling. After excluding questionnaires with incomplete and regular responses, the number of valid questionnaires with no missing values was 752 (66.4% female). The average age of all the participants was 19.36 years (*SD* = 1.24), with an age range from 17 to 23 years old. Two hundred and fifty-six (34.0%) of them are freshmen, two hundred and seventy-two (36.2%) of them are sophomores, two hundred and twenty-one (29.8%) of them are juniors. After the participants comprehended the requirements of the survey, they were conducted in the form of paper-and-pencil within 30 min in classrooms. The study had been approved by the Ethics Committee for Scientific Research at the corresponding author’s unit before the survey was conducted.

### 2.2 Measurements

#### 2.2.1 Mobile phone addiction

The Mobile Phone Addiction Index (MPAI; 42) was utilized in this study and had shown good reliability and validity among Chinese college students (43). The scale comprised 17 items and assessed 4 factors connected with mobile phone addiction including inability to control cravings, anxiety and feeling lost, withdrawal and escape, as well as productivity loss (e.g., “Someone said that you spend too much time on your mobile phone”). All items were scored by using Likert-type scale ranging from 1 (never) to 5(always). All scores were added together and averaged, with higher scores reflecting greater mobile phone addiction. Cronbach’s α for this study was 0.84.

#### 2.2.2 Technology conflict

The technology conflict scale (26) was adapted to assess two factors including technology-personal conflict, and technology-academic conflict (e.g., “Mobile phone usage influences my school work/work”). The reliability and validity of the Chinese version of this questionnaire have been recognized (26). Participants rated 6 items on a seven-point scale, ranging from 1 (strongly agree) to 7 (strongly disagree). All scores were added together and averaged, with higher scores indicating higher levels of technology conflict. Cronbach’s α for this scale was 0.82 in this study.

#### 2.2.3 Mindfulness

Chinese version of mindfulness scale was employed to assess the level of participants’ mindfulness and had shown good reliability and validity among Chinese college students (10). The scale included ten items that were responded to participants invited on a scale from 0 (never) to 4 (always) (e.g., “I am depressed for unreasonable feelings.”). The average of all items’ score was used to represent the level of mindfulness. Cronbach’s α was 0.71 in the current study.

#### 2.2.4 Academic burnout

Maslach Burnout Inventory-Student Survey (11) translated into Chinese version was assessed in this study. Participants responded to the 15 items on a Likert-type scale ranging from 1 (not true) to 7 (extremely true) (e.g., “Studying makes me feel exhausted”). The average of responses is to measure students’ academic burnout, with higher scores representing higher levels of academic burnout. Cronbach’s α for this scale was 0.84.

#### 2.2.5 Control variables

Gender, age, grade and the years of using mobile phone were included as the control variables in this study, as prior studies proved that the observed variables of this study have significant difference from gender, age, grade and the years of using mobile phone (44–46).

### 2.3 Statistical analyses

Descriptive statistics and Pearson correlations were utilized to calculate averages, standard deviations, and exploring bivariate associations for the variables under observation in our study, employing SPSS 23.0. Subsequently, the SPSS macro PROCESS (model 8), as developed by Hayes (47), was employed to assess the proposed moderated mediation model. This SPSS macro has demonstrated enhanced statistical testability in examining mediating and moderating models across various studies. Finally, to gain a more in-depth understanding of the significant interaction effects, we conducted simple slopes analyses, employing the approach outlined by Toothaker (48). This process involved decomposing the interactions to elucidate the specific nuances and variations in the relationship between mobile phone addiction, technology conflict mindfulness, and academic burnout. By employing these analytical techniques, our study aims to provide a robust and nuanced examination of the interplay between these variables, shedding light on the potential moderating and mediating mechanisms at play.

## 3 Results

### 3.1 Preliminary analyses

**Table 1** presented the average, standard deviations, and correlations of the all observed variables. The average mobile phone addiction was 2.612 (*SD* = 0.598), with range from 1 to 4.71. As what we had hypothesized, mobile phone addiction was positively connected with technology conflict (*r* = 0.447, *p* < 0.001) and academic burnout (*r* = 0.301, *p* < 0.01) while had a negative connection with mindfulness (*r* = -0.449, *p* < 0.001). Technology conflict was positively related with academic burnout (*r* = 0.330, *p* < 0.001) and negatively correlated with mindfulness (*r* = -0.409, *p* < 0.001). Mindfulness was negatively related with academic burnout (*r* = -0.324, *p* < 0.001).

**Table 1:** Descriptive statistics and interrelations among all of the observed variables.

Variables	*M*	*SD*	1	2	3	4	5	6	7	8
1. Gender	1.66	0.473	1							
2. Age	19.36	1.242	-0.017	1						
3. Grade	1.96	0.798	0.019	0.144^**^	1					
4. Years of using mobile phones	4.71	2.252	0.035	0.003	0.049	1				
5. Mobile phone addiction	2.612	0.598	0.029	-0.068	-0.073^*^	0.031	1			
6. Technology conflict	4.182	1.179	0.036	-0.043	-0.037	0.014	0.447^***^	1		
7. Mindfulness	2.109	0.453	-0.002	0.020	0.038	0.014	-0.449^***^	-0.409^***^	1	
8. Academic burnout	2.301	0.282	-0.002	0.005	-0.019	0.012	0.301^**^	0.330^***^	-0.324^***^	1

N =752. *p < 0.05. **p < 0.01. ***p < 0.001.

### 3.2 Testing for the proposed moderated mediation model

The adapted SPSS macro PROCESS (47) was employed to scrutinize the proposed moderated mediation model, and the key findings are summarized in **Table 2**. This table encompassed the total effect model (model 1), the mediator variable model (model 2), and the dependent variable model (model 3), along with the conditional direct effect analysis. In particular, model 1 assessed the overall impact of mobile phone addiction on academic burnout. Model 2 explored the effects of mobile phone addiction, mindfulness, and their interactions on technology conflict. Model 3 delved into the effects of mobile phone addiction, technology conflict, mindfulness, and the interactions of mobile phone addiction and mindfulness on academic burnout. Lastly, two conditional effect analyses were conducted to illustrate the distinct direct and indirect effects of mobile phone addiction on academic burnout at different levels of mindfulness.

**Table 2:** Regression results for the conditional direct and indirect effects.

Model: Total effect model									
R	R^2^	F	df_1_	df_2_	p	β	SE	t	p
0.301	0.091	14.012	5	746	<0.001				
Constant						-0.539	0.747	-0.722	>0.05
Gender						0.026	0.073	0.035	>0.05
Age						0.030	0.043	0.701	>0.05
Grade						-0.036	0.064	-0.571	>0.05
Years of using mobile phones						-0.004	0.018	-0.205	>0.05
Mobile phone addiction						0.301^***^	0.038	8.029	<0.001
Model: Mediator variable model									
R	R^2^	F	df_1_	df_2_	p	β	SE	t	p
0.516	0.266	36.266	7	744	<0.001				
Constant						-0.193	0.779	-0.247	>0.05
Gender						0.103	0.070	1.474	>0.05
Age						0.002	0.044	0.044	>0.05
Grade						-0.002	0.068	-0.031	>0.05
The years of using mobile phone						-0.012	0.015	-0.797	>0.05
Mobile phone addiction						0.314^***^	0.036	8.823	<0.001
Mindfulness						-0.245^***^	0.037	-6.610	<0.001
Mobile phone addiction × Mindfulness						-0.101^**^	0.030	-3.329	<0.01
Model: Dependent variable model									
R	R^2^	F	df_1_	df_2_	p	β	SE	t	p
0.414	0.171	20.653	8	743	<0.001				
Constant						-0.691	0.714	-0.968	>0.05
Gender						0.022	0.069	0.316	>0.05
Age						0.036	0.041	0.873	>0.05
Grade						-0.043	0.060	-0.719	>0.05
The years of using mobile phone						0.004	0.017	0.219	>0.05
Technology conflict						0.185^***^	0.038	4.938	<0.001
Mobile phone addiction						0.119^**^	0.044	2.704	<0.01
Mindfulness						-0.180^***^	0.041	-4.396	<0.001
Mobile phone addiction × Mindfulness						-0.075^*^	0.029	-2.588	<0.05
Conditional direct effect analysis at values of mindfulness (M ± SD)						β	Boot SE	Boot LLCI	Boot ULCI
*M* − 1*SD* (1.656)						0.194	0.052	0.092	0.296
*M* (2.109)						0.119	0.044	0.033	0.205
*M* + 1*SD* (2.462)						0.044	0.054	-0.061	0.149
Conditional indirect effect analysis at values of mindfulness (M ± SD)						β	Boot SE	Boot LLCI	Boot ULCI
*M* − 1*SD* (1.656)						0.077	0.017	0.045	0.113
*M* (2.109)						0.058	0.013	0.035	0.087
*M* + 1*SD* (2.462)						0.039	0.012	0.020	0.069

N = 752. Standardized regression coefficients are reported. Bootstrap sample size = 5000. LL = low limit, CI = confidence interval, UL = upper limit. *p < 0.05. **p < 0.01. ***p < 0.001.

As anticipated, the total effect model [*F* (5, 746) = 14.012, *R^2 =^* 0.091, *p* < 0.001], the mediator variable model (*F* (7, 744) = 36.266, *R^2 =^* 0.266, *p* < 0.001), and the dependent variable model [*F* (8, 743) = 20.653, *R^2 =^* 0.171, *p* < 0.001] were all significant, even after controlling for gender, age, grade, and years of mobile phone usage. Specifically, mobile phone addiction positively predicted technology conflict (*β* = 0.314, *p* < 0.001) and academic burnout (*β* = 0.119, *p* < 0.001). Technology conflict positively predicted academic burnout (*β* = 0.185, *p* < 0.001). Besides, the mediating effect of technology conflict was 0.109, accounting for 36.21% of the total effect of mobile phone addiction on academic burnout (0.301). This study’s results robustly demonstrated the connection between mobile phone addiction, academic burnout, and the mediating role of technology conflict, thus supporting Hypotheses 1 and 2.

**Figure 2:**
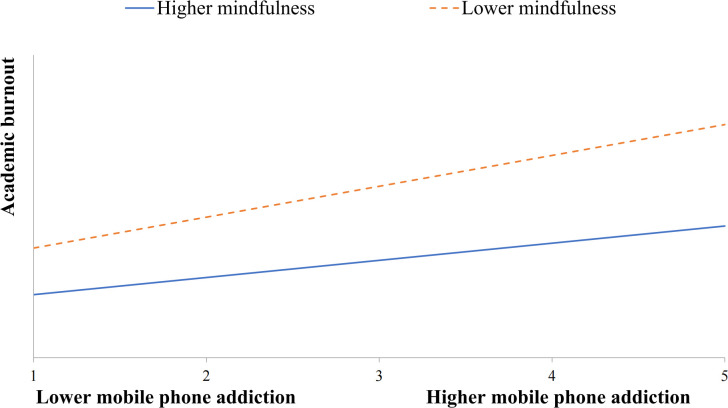
Mindfulness moderates the relation between mobile phone addiction and academic burnout.

**Figure 3:**
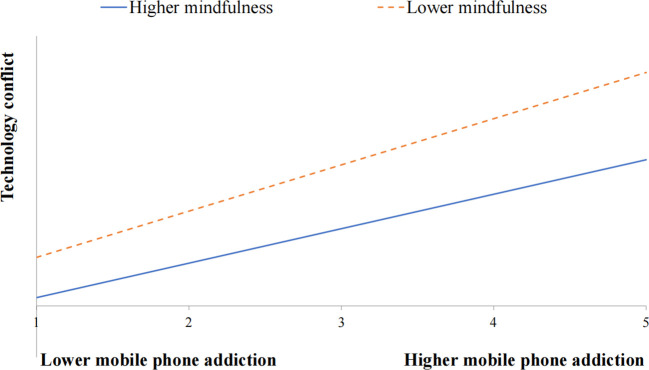
Mindfulness moderates the relation between mobile phone addiction and technology conflict.

Furthermore, a significant interaction effect was observed between mobile phone addiction and mindfulness on technology conflict (*β* = -0.101, *p* < 0.01) and academic burnout (*β* = -0.075, *p* < 0.05). This indicated that the relationship between mobile phone addiction and both technology conflict and academic burnout was moderated by mindfulness. Simple slope analyses were employed to deconstruct these interactions, revealing differences between slopes for the high mindfulness group (1 *SD* above the mean) and the low mindfulness group (1 *SD* below the mean). **Figures 2**, **3** visually depict these results. In **Figure 2**, the effect of mobile phone addiction on academic burnout was not significant for students with high levels of mindfulness (*β* =0.085, *t* = 1.609, *p* > 0.05), while it was significant for students with low levels of mindfulness (*β* =0.153, *t* =3.805, *p* < 0.001). **Figure 3** illustrated that the effect of mobile phone addiction on technology conflict was significant for students with high levels of mindfulness (*β* =0.268, *t* = 6.558, *p* < 0.001) as well as those with low levels of mindfulness (*β* =0.340, *t* =12.838, *p* < 0.001). Specifically, students with lower levels of mindfulness reported higher levels of technology conflict and academic burnout than their counterparts with higher levels of mindfulness when faced with mobile phone addiction. In order to analyze the specific effect sizes of the above moderating effects, the Johnson-Neyman technique was conducted using the PROCESS program. The Johnson-Neyman analysis showed that mobile phone addiction was a significant predictor of academic burnout when mindfulness was less than 2.485. The predictive effect of mobile phone addiction on technology conflict was significant when mindfulness was less than 2.908.

Additionally, all of the conditional direct effects and indirect effects have significant difference from zero according to the result of the condition analyses. In other words, both the direct effect of mobile phone addiction on academic burnout and the indirect effect of mobile phone addiction on academic burnout through technology conflict were stronger for the lower-mindfulness group. Thus, hypothesis 3 was supported.

## 4 Discussion

With the rapid advancement of Internet technology, mobile phones have gradually become increasingly significant in the study and life of college students (49). As a result, more and more college students were confronting the threat of mobile phone addiction (50). This study was designed to explore both the mediating mechanisms (how) and the moderating mechanisms (when or for whom) through which mobile phone addiction contributed to college students’ academic burnout. The findings confirmed Hypothesis 1, indicating a positive association between mobile phone addiction and academic burnout. Moreover, the results of moderated mediation analyses supported Hypothesis 2, revealing that technology conflict mediated the relationship between mobile phone addiction and academic burnout. Additionally, Hypothesis 3 was confirmed, signifying that mindfulness acted as a buffering factor, mitigating the impact of mobile phone addiction on academic burnout and technology conflict. These results suggested that mindfulness training could potentially reduce the negative consequences of mobile phone addiction on college students’ mental health and behavior adaptation.

First, in line with previous research, this study affirmed that mobile phone addiction significantly and positively predicted college students’ academic burnout (51). While mobile phone use could assist students in accessing a wealth of learning resources to meet various educational needs, excessive use could lead to detrimental consequences. Unrestrained mobile phone use may impair individuals’ ability to control attention. As a result, they may experience difficulty concentrating while completing their study tasks, increasing the likelihood of academic burnout (8, 29). In addition, studies indicated that mobile phone addiction has a notable impact on an individual’s self-concept clarity (52). Those with a blurred self-concept lacked a defined plan for the future, exhibited reduced motivation for learning (53), and consequently may be more susceptible to experiencing academic burnout.

Second, the results of this study demonstrated that technology conflict acted as a mediator between mobile phone addiction and academic burnout among college students. Previous studies have revealed that technology conflict was an undesirable consequence of excessive technological equipment use (e.g., mobile phone addiction) (26). Excessive mobile phone use could trigger cognitive preoccupations, which may have a negative impact on an individuals’ physical and mental health. Therefore, technology conflict may be a bridge between excessive technological device use and academic adjustment among college students. Building on previous research, this study revealed the mediating role of technology conflict in mobile phone addiction and academic burnout from the more nuanced perspectives of technology-personal conflict and technology-academic conflict. Specifically, individuals with mobile phone addiction were prone to experiencing negative emotions like depression and anxiety (3). Simultaneously, they may suffer from health problems such as sedentary back pain (33). Consequently, technology-personal conflict, as a vital dimension of technology conflict, amplified the sense of resource loss in students, thereby triggering academic burnout. Additionally, this conclusion aligns with the cognitive behavior model (54), which posits that individuals with mobile phone addiction showed fundamental cognitive dysfunction, leading to various behavioral issues, including academic burnout. Moreover, mobile phone addiction diminished impulse control, fostering cognitive distortions about themselves. This negative self-perception could decrease academic self-efficacy, hamper learning motivation, ultimately fueling technology-academic conflict. Thus, the technology conflict induced by mobile phone addiction unfolds as a cascade of academic problems, particularly academic burnout. In summary, technology conflict served as a potential mechanism to comprehend how mobile phone addiction induced academic burnout. Therefore, understanding the mediating role of technology conflict could help to block the causal chain of mobile phone addiction leading to academic burnout from the perspective of intervening in technology conflict, such as outdoor exercise, and appropriate exercise to mitigate technology-personal conflict.

Moreover, a crucial finding in this study pertained to the individual differences in the predictive effects of mobile phone addiction on technology conflict and academic burnout. Specifically, mindfulness emerged as a moderator, influencing both the direct effect of mobile phone addiction on academic burnout and the indirect effect through technology conflict. These results highlighted the role of mindfulness as a buffer, contributing to the maintenance of mental health, emotional maturity, and the alleviation of potential adverse effects of mobile phone addiction on physical and mental adaptation, as well as academic performance.

The moderating effects of mindfulness on the relationship between mobile phone addiction and its consequences (academic burnout and technology conflict) could be clarified through several mechanisms. Firstly, the re-perceiving model of mindfulness proposed that engaging in mindfulness practices enabled individuals to focus on their moment-to-moment experiences with heightened objectivity and awareness (55). Consequently, individuals with high levels of mindfulness were more inclined to direct their attention to their studies, thereby minimizing the adverse impacts of mobile phone addiction. Moreover, mindfulness has been linked to increased self-esteem, resilience, optimism, and hope (56–58), all of which are crucial psychological resources (59). Following the model of conservation of resources (60), individuals strive to preserve, protect, and build valued resources. Having enhanced psychological resources equips individuals with a greater capacity to cope with stress. Therefore, individuals with high levels of mindfulness may exhibit more effective coping mechanisms when confronted with mobile phone addiction and its negative consequences. Furthermore, the mindfulness stress-buffering hypothesis posits that mindfulness could alleviate the detrimental impacts of stress on psychological and physical adaptation (61, 62). In this context, mindfulness may attenuate the association between negative factors, such as mobile phone addiction, and individuals’ psychological and physical adaptation problems (e.g., academic burnout and technology conflict). Consequently, college students with high levels of mindfulness were less likely to succumb to technology conflict and academic burnout when grappling with mobile phone addiction. The interplay between mindfulness and mobile phone addiction underscores the protective role of mindfulness in mitigating the potential negative outcomes associated with excessive technology use, thereby contributing to a more resilient and adaptive response among college students. This study further elaborated the protective effects of mindfulness as a positive psychological trait for individuals, enriching the empirical research on the protective effects of mindfulness. Therefore, mindfulness interventions for individuals with mobile phone addiction may be able to reduce their technology conflict and academic burnout.

## 5 Limitations and implications

Despite the valuable insights provided by this study regarding the mediation and moderation mechanisms between mobile phone addiction and academic burnout, certain limitations should be acknowledged.

First, the study focused exclusively on a sample of college students in China, and the generalization of the findings to students from other cultural backgrounds may be limited. Cultural variations in the values and effects of mindfulness exist, and future research should explore diverse cultural contexts to enhance the universality of the results. While existing studies have indicated positive outcomes related to mindfulness in the academic field across various cultures (63–65), additional cross-cultural investigations are needed to validate these findings. Additionally, the study’s reliance on a relatively small sample of college students warrants further research with larger and more diverse samples, encompassing different age groups to enhance the generalizability of the results. Second, due to the cross-sectional design, this study did not establish a strict causal relationship between variables. Future research could employ a longitudinal approach to track the development of mobile phone addiction, technology conflict, and academic burnout over time, shedding light on the temporal dynamics and causal connections between these variables. Intervention studies aimed at improving mindfulness levels among individuals with mobile phone addiction could further elucidate the buffering effect of mindfulness on technology conflict and academic burnout. Moreover, potential biases, such as social desirability bias and common method bias, may be present due to the reliance on self-report questionnaires. Future research could explore alternative methods, such as multidimensional scaling, and incorporate objective data from diverse perspectives to minimize biases.

Notwithstanding these limitations, the study contributed to the existing literature by elucidating the mediating role of technology conflict and the protective role of mindfulness, offering insights into how and when mobile phone addiction contributed to academic burnout. Furthermore, the study held practical significance, particularly for college students who were increasingly immersed in mobile phones, potentially facing irrational procrastination and various psychological adaptation challenges. The findings underscore the importance of guiding students to maintain a balanced relationship with mobile phones and adopt positive self-awareness. Improving mindfulness levels among students could be a meaningful intervention, aiding in consciously responding to excessive mobile phone use and mitigating its negative effects. Mindfulness training, including practices such as yoga and mindfulness listening, has proven effective in enhancing self-control, providing a potential avenue for reducing the adverse impacts of uncontrolled mobile phone use. In conclusion, college students, particularly those with low levels of mindfulness, could benefit from mindfulness improvement attempts. These included paying attention to body posture, thoughts, emotions, and activities, avoiding self-evaluation and comparison, staying aware of current existence, and embracing differences in physiological and psychological states. Additionally, addressing technology conflict as a mediator in the relationship between mobile phone addiction and academic burnout could be accomplished by implementing strategies to change cognitive preemption and emotional responses. Developing study plans, controlling internet usage time, viewing mobile phones as tools rather than life’s protagonists, cultivating hobbies, and making rational use of spare time were suggested approaches to reduce technology conflicts and foster a healthier learning state among college students.

## Data availability statement

The original contributions presented in the study are included in the article/**Supplementary Material**. Further inquiries can be directed to the corresponding authors.

## Ethics statement

The studies involving humans were approved by AnYang Normal University, Ethic Committee, EC, Institutional Review Board. The studies were conducted in accordance with the local legislation and institutional requirements. Written informed consent for participation in this study was provided by the participants’ legal guardians/next of kin.

## Author contributions

G-HY: Conceptualization, Formal analysis, Writing – original draft, Writing – review & editing. X-XC: Conceptualization, Formal analysis, Writing – original draft, Writing – review & editing. Y-YF: Writing – original draft, Data curation. N-DW: Methodology, Supervision, Writing – review & editing. S-LL: Project administration, Supervision, Writing – review & editing.
